# *gltB* encoding glutamate synthase is involved in persister and biofilm formation and virulence in *Staphylococcus aureus*

**DOI:** 10.1128/spectrum.00511-25

**Published:** 2025-08-12

**Authors:** Xueyi Wen, Tian Tang, Tingrui Bao, Tao Xu, Bei An, Ying Zhang, Jian Han

**Affiliations:** 1 Department of Pathogenic Biology, School of Basic Medical Sciences, Lanzhou University 12426, Lanzhou, Gansu, China; 2 Department of Infectious Diseases, Shanghai Key Laboratory of Infectious Diseases and Biosafety Emergency Response, National Medical Center for Infectious Diseases, Huashan Hospital, State Key Laboratory of Genetic Engineering, School of Life Science, Fudan University 12478, Shanghai, China; 3 State Key Laboratory for the Diagnosis and Treatment of Infectious Diseases, The First Affiliated Hospital, Zhejiang University School of Medicine 26441 , Hangzhou, Zhejiang, China; 4 Jinan Microecological Biomedicine Shandong Laboratory 661980 , Jinan, Shandong, China; University of Pittsburgh, Pittsburgh, Pennsylvania, USA

**Keywords:** *Staphylococcus aureus*, *gltB*, glutamate metabolism, persisters, biofilms, virulence, persistent infections

## Abstract

Glutamate metabolism plays a pivotal role in linking the tricarboxylic acid cycle, arginine biosynthesis, and purine metabolism, and these pathways have been shown to be involved in persister formation. However, the relationship among glutamate metabolism, bacterial antibiotic tolerance, and virulence remains unclear. In this study, *gltB,* which encodes the large subunit of glutamate synthase in *Staphylococcus aureus,* was knocked out. The ∆*gltB* mutant in the stationary phase showed less tolerance to antibiotics and was killed completely after exposure to lethal doses of ampicillin and norfloxacin after 11 and 6 days, respectively, while the parent strain still had abundant viable bacteria. The *gltB* complemented strain restored antibiotic tolerance. Interestingly, exogenous glutamate supplementation of ∆*gltB* restored the tolerance to antibiotics. Moreover, ∆*gltB* is more susceptible to heat, carbon starvation, and oxidative stress. Furthermore, the ability of ∆*gltB* to coagulate plasma, produce staphyloxanthin, and form biofilms was significantly weakened. In addition, ∆*gltB* attenuated virulence in BALB/c mice, and its 50% lethal dose (LD50) (1.14 × 10^10^ CFU/mL, 95% CI: 7.29 × 10^9^–2.75 × 10^10^) was higher than that of the parent strain (2.39 × 10^9^ CFU/mL, 95% CI: 9.99 × 10^8^–4.42 × 10^9^). The expression levels of major virulence genes, including *eta, hla, hlgA, hlgC, lukD, lukE, lukS, lukF,* and *sea,* as well as staphyloxanthin synthesis-related genes, including *crtM* and c*rtQ,* were significantly downregulated in ∆*gltB*. This study revealed that *gltB* is involved in both antibiotic tolerance and virulence in *S. aureus* and provides new insights into the mechanism of persister formation and virulence, with implications for the development of novel drugs.

**IMPORTANCE:**

*Staphylococcus aureus* is a leading bacterial cause of death, and persister formation renders it tolerant to antibiotics and is associated with its persistent infections. Glutamate metabolism plays a critical role in linking the tricarboxylic acid cycle, arginine biosynthesis, and purine metabolism, and these pathways have been shown to be involved in persister formation. This work first discovered that *gltB*, the large subunit of glutamate synthase gene in *S. aureus*, is involved in tolerance to antibiotics and heat, carbon starvation, and oxidative stress. Furthermore, the *gltB* mutant attenuated virulence in mice, owing to the inhibition of glutamate synthesis, which significantly weakened the ability of *S. aureus* to coagulate plasma, produce staphyloxanthin, form biofilms, and express virulence factors. These findings confirm the important role of glutamate metabolism in the formation of persister and virulence in *S. aureus* and provide new targets for developing novel anti-persister and anti-virulence drugs.

## INTRODUCTION

Persisters were first described by Joseph Bigger in 1944 when he found that *Staphylococcus aureus* treated with high doses of penicillin retained a small number of surviving cells (1). Persisters are dormant cells with a low metabolism (2, 3). Unlike resistant bacteria, persister cells are non-heritable phenotypic variants, and the regrowth population remains susceptible to the same antibiotic (2, 4, 5). Persisters present a significant challenge to effective antimicrobial therapies and pose significant public health challenges as they contribute to treatment failure, prolonged treatment, and chronic and relapsing infections (6–8). Furthermore, evidence suggests that persistence promotes the development of genetic antibiotic resistance (7, 9).

Persisters are found in all bacteria, including a variety of bacterial pathogens, both *in vitro* and *in vivo*, such as *S. aureus*, *Escherichia coli*, *Pseudomonas aeruginosa*, and *Mycobacterium tuberculosis* (2, 3). Several factors are thought to contribute to the development of persister cells. Altered energy production is a commonly accepted theory that affects bacterial persistence (10–14). Toxin-antitoxin modules play a role in persister formation in *E. coli* (15). Blocking DNA replication or transcription, which inhibits growth, increases the number of persister cells (16, 17). For example, increased alarmone (p)ppGpp levels can lead to persister cells in *E. coli*, which inhibit negative supercoiling of DNA and prevent DNA replication and transcription, resulting in bacterial persistence (16). Furthermore, repressing protein translation is also a generally acknowledged mechanism of persister formation (18–20). These studies commonly used *E. coli* as a model. However, the mechanisms of persister formation are disparate in different microorganisms, though their mechanisms of persister formation have similar pathways in terms of functions due to convergent evolution (3).

*S. aureus* is the leading bacterial cause of death in 135 countries, which can cause multiple infections, ranging from skin and soft tissue infections to life-threatening infections, such as sepsis and endocarditis (21, 22). Persister formation in *S. aureus* renders it tolerant to high concentrations of antibiotics and is associated with chronic and recurrent infections. In recent years, significant progress has been made in understanding the mechanisms of persister formation in *S. aureus*. Multiple genes, including *glpF, sdhA/sdhB, agrCA/agrD*, *msaB*, *mprF*, *ctaB*, *clpX, clpC,* and *clpP*, has been shown to be involved in the persistence of *S. aureus* (11, 14, 23–29). Moreover, ATP levels also affect persister levels in *S. aureus* (13), and changes in energy metabolism, such as tricarboxylic acid (TCA) cycle gene expression, could affect persister formation (11, 30). *argJ,* involved in arginine biosynthesis, has been shown to be critical for persister formation in *S. aureus* (27). *purB, purM,* and *purN* genes, which participate in purine metabolism, play important roles in *S. aureus* persistence (31, 32).

Despite these advances in understanding the mechanisms of persister formation in *S. aureus*, many questions remain. Multiple pathways are involved in persister formation in *S. aureus*, but how these pathways coordinate with each other is worth further investigation. Kyoto Encyclopedia of Genes and Genomes (KEGG) pathway analysis revealed that glutamate metabolism could link the TCA cycle, arginine biosynthesis, and purine metabolism, which are involved in persister formation. Studies have shown that mutations in *gltI* and *gltT* genes, which are involved in glutamate transport, affect persister formation in *E. coli* and *S. aureus* (33, 34), suggesting that glutamate may participate in persister formation in bacteria. However, the relationship among glutamate metabolism, bacterial antibiotic tolerance, and virulence remains unclear. *gltB*, which encodes a large subunit of glutamate synthase, is a key gene in *S. aureus* and participates in the alanine, aspartate, and glutamate metabolic pathways (35, 36). In this study, we show that *gltB* is responsible for persister formation in *S. aureus* and is closely related to bacterial pigment formation, biofilm formation, and virulence. Our findings provide new insights into the mechanisms underlying persister formation and have implications for the development of novel anti-persister and anti-virulence drugs.

## MATERIALS AND METHODS

### Bacterial strain, culture conditions, and antibiotics

*S. aureus* Newman strain obtained from the American Type Culture Collection (ATCC 700926) was used to construct a *gltB* allelic replacement mutant (∆*gltB*) and to perform persister assays, pigment, and virulence tests. Newman::pRAB11, ∆*gltB*::pRAB11, and ∆*gltB*::pRB*gltB* were used to perform the persister and biofilm assays. *E. coli* DC10B was used to construct the recombination shuttle plasmids, pMX10-*gltB* and pRAB-*gltB*. Tryptic Soy Agar (TSA) and Trypticase Soy Broth (TSB) (Oxoid, England) were used to culture *S. aureus,* and LB medium (Solarbio, Beijing, China) was used to culture *E. coli*. The antibiotics ampicillin, norfloxacin, levofloxacin, vancomycin, rifampin, anhydrotetracycline (Atc), and chloramphenicol were obtained from Sangon Biotech (Shanghai) Co., Ltd. (Shanghai, China). Ampicillin and norfloxacin/levofloxacin were used in persister assays at a concentration of 20 µg/mL. Chloramphenicol (10 µg/mL) was used for the induction and screening of the ∆*gltB* mutant by homologous recombination, and Atc (100 ng/mL) was used to induce pRAB-*gltB* expression.

### Construction of an allelic replacement mutant and its complemented strain of *S. aureus*

The primers, including gltB-uf-KpnI, gltB-ur, gltB-df, and gltB-dr-EcoRI, used to knock out *gltB* of the *S. aureus* Newman strain are listed in Table S1. Two DNA fragments besides *gltB* were amplified and fused into one fragment using overlap PCR. pMX-*gltB* was constructed by inserting the fused DNA fragment into the pMX10 vector (29). After pMX-*gltB* was transformed into *E. coli* DC10B and confirmed to be the correct recombinant plasmid, the shuttle plasmid was transformed into *S. aureus* Newman competent cells by electroporation as described previously (14). The transformed *S. aureus* was selected, and ∆*gltB* was obtained according to the standard homologous recombination procedure (37) and verified by DNA sequencing.

To construct the complementation strain ∆*gltB*::pRAB*gltB*, the genomic DNA of the Newman strain was used as a template, and wild-type *gltB* was amplified using primers gltB-f and gltB-r (Table S1). The PCR product was digested with *BgI*II and *Eco*RI, and then ligated into the plasmid pRAB11. The recombinant plasmid, pRAB-*gltB,* was transformed into *E. coli* DC10B and confirmed by sequencing. The shuttle plasmid was transformed into ∆*gltB* competent cells via electroporation. The complemented strain ∆*gltB*::pRAB*gltB* was induced by Atc, and the expression level of *gltB* was confirmed by quantitative reverse transcription PCR (qRT-PCR).

### Minimum inhibitory concentration and minimum bactericidal concentration determination for the wild-type and the Δ*gltB* mutant

Minimum inhibitory concentration (MIC), which was recorded as the minimum drug concentration that inhibited visible growth of the *S. aureus* Newman wild-type and the Δ*gltB* mutant, was determined by serial twofold dilutions of the antibacterial agents (including ampicillin, norfloxacin, vancomycin, and rifampin) in TSB in a 96-well plate. The number of viable bacteria before and after treatment with the drugs in each well was counted, and the minimum drug concentration that killed 99.9% of the initial inoculated bacteria was defined as the minimum bactericidal concentration (MBC).

### Persister assay

Overnight *S. aureus* and the mutant cultures were diluted 1:1,000 fold by TSB in culture tubes, and the subcultures were incubated at 37°C with 180 rpm shaking. Following incubation for designated time intervals and initial bacterial quantification through serial dilution, the survival of *S. aureus* upon exposure to ampicillin (20 µg/mL) and norfloxacin (20 µg/mL) was evaluated using antibiotic exposure assays. The cultures exposed to antibiotics were incubated at 37°C without shaking, and 100 µL of the cultures exposed to drugs was collected daily, washed in TSB two times, and plated for CFU determination on TSA plates after appropriate dilutions.

For the persister assay after supplementation with exogenous glutamate, overnight *S. aureus* and ∆*gltB* cultures were diluted 1:1,000 fold by TSB supplemented with glutamate (10 mM) and incubated at 37°C with 180 rpm shaking. After 48 h of incubation, the survival of *S. aureus* upon exposure to ampicillin (20 µg/mL) and levofloxacin (20 µg/mL) was evaluated using antibiotic exposure and CFU assays.

### Exposure assays for the detection of susceptibility to various stresses

Overnight WT::pRAB11, Δ*gltB*::pRAB11, and Δ*gltB*::pRAB*gltB* cultures were diluted 1:1,000 fold with TSB containing Atc (100 ng/mL), and the subcultures were incubated for 24 h at 37°C with 180 rpm shaking. These cultures were used to perform exposure assays to detect susceptibility to various stresses. For tolerance to heat, 1 mL of each subculture was transferred into an Eppendorf tube and placed in a water bath at 56°C ± 0.1°C. After 30, 60, 90, and 120 min of incubation, the cultures were diluted with TSB, and the survival of bacteria was determined on TSA plates. For carbon starvation, 1 mL of each culture was washed thrice with PBS and resuspended in PBS_Atc0.1_. The cell suspensions were incubated at 37°C without shaking, and the viable bacteria in the suspensions were determined after 3, 5, 7, 10, 13, 15, 17, and 20 days of incubation. For oxidative stress tests, 1 mL of the cultures was washed once with PBS and resuspended in PBS_Atc0.1_. An equal amount of 5% H_2_O_2_ was added to the suspensions, which were incubated at 37°C without shaking, and the viable bacteria in the suspensions were determined after 30 and 60 min incubation by CFU count.

### Detection of staphyloxanthin production

Overnight cultures of the *S. aureus* Newman wild-type and the Δ*gltB* mutant were inoculated into TSA. After incubation for 24 h at 37°C, the size, shape, and color of the colonies on the plates were observed. In addition, overnight cultures of WT::pRAB11, Δ*gltB*::pRAB11, and Δ*gltB*::pRAB*gltB* were inoculated into TSB_Atc0.1_. After incubation for 6, 12, 24, 48, and 72 h, bacterial cells were collected by centrifugation and washed once with ddH_2_O. The color of the precipitate was observed. The bacterial pellets were resuspended in 1 mL of methanol. After incubation at 55°C for 20 min and centrifugation at 15,000 rpm for 5 min, the supernatants were transferred to a 96-well plate, and the OD_462_ of the samples was determined using a microplate reader.

### Detection of coagulase activity of *S*. *aureus*

One hundred microliters of rabbit plasma containing sodium citrate were dropped onto a slide. Twenty microliters of overnight cultures of the wild-type and the Δ*gltB* mutant in TSB were pipetted and mixed thoroughly in rabbit plasma. Coagulated particles formed in the mixture were observed after standing for 10 min at room temperature.

### Biofilm formation ability of bacteria

The ability of the *S. aureus* strains to form biofilms was determined in 96-well plates, with modifications to a previously published method (38). After 200 µL of TSB containing 0.25% glucose, 10 µg/mL chloramphenicol, and 0.1 µg/mL Atc was added to the corresponding wells of 96-well plates, 1 µL of overnight cultures of WT::pRAB11, Δ*gltB*::pRAB11, and Δ*gltB*::pRAB*gltB* was inoculated into the corresponding wells. The plate was incubated for 24 h at 37°C without shaking, and then the supernatants in the wells were removed, and the wells were washed three times with PBS. After fixing the biofilms at 65°C for 45 min, 200 µL of 0.5% crystal violet solution was added to the wells and stained at room temperature for 25 min. After the wells were washed with water, the biofilms formed in each well were photographed, and 100 µL of 33% acetic acid was added to the wells to dissolve the crystal violet. Relative biofilm formation was assayed by measuring optical density at 570 nm.

### 50% lethal dose of the wild-type and the Δ*gltB* mutant in BALB/c mice

The 50% lethal dose (LD50) of the wild-type and the Δ*gltB* mutant in BALB/c mice was assessed in 8-week-old female BALB/c mice (purchased from Experimental Animal Center of Lanzhou University) based on the reported literature (32). Seventy-five female BALB/c mice were randomly divided into 15 subgroups (five mice in each subgroup). Then, a subgroup was selected randomly as the PBS group, and the other 14 subgroups were randomly divided into the WT group and the Δ*gltB* group. Each subgroup in the WT group and the Δ*gltB* group was randomly divided into seven different grades based on the injected bacterial dose. A random-number table was used to generate the randomization sequence. Overnight cultures of the *S. aureus* Newman wild-type and the Δ*gltB* mutant were washed twice with PBS, concentrated with PBS for 10-fold, and then the bacterial suspensions of each strain were subjected to twofold serial dilutions to form seven different gradients. The bacterial suspension was intraperitoneally injected into mice at 0.6 mL per mouse. The injected mice were raised normally, and their activity and dietary status were observed daily. The survival and death status of mice were recorded in each group for 7 consecutive days, and the LD50 and 95% confidence intervals (CIs) were calculated by Probit analysis.

### RNA isolation

Overnight cultures of *S. aureus* were collected by centrifugation and stored at −80°C. A 50 U of lysostaphin (Shanghai Hi-Tech Bioengineering Co., Ltd., China) was added to the bacterial suspension and incubated at 37°C for 45 min. After the cells were treated with 1 mL of Trizol and 200 µL of chloroform, an equal volume of pre-cooled isopropanol was added. After centrifugation for 10 min at 12,000 × *g* and 4°C, the precipitate was washed once with pre-cooled ethanol and centrifuged for 5 min at 7,500 × *g*. The precipitate was dissolved in 40 µL of RNase-free water. The concentration of isolated RNA was quantified using a NanoDrop (Thermo Scientific, USA). Total RNA was stored at −80°C. Each sample was prepared in triplicate.

### Quantitative reverse transcription PCR

The expression levels of *gltB* in Newman::pRAB11 and ∆*gltB*::pRAB*gltB*, virulence genes, including *eta*, *hla*, *hlgA*, *hlgB*, *hlgC*, *lukD*, *lukE*, *lukS*, *lukF*, and *sea*, and staphyloxanthin-related genes, including *mvaS*, *crtM,* and *crtQ,* were detected by qRT-PCR. Primers corresponding to the genes were designed using NCBI Primer-BLAST, and the sequences are displayed in Table S1.

For qRT-PCR, total RNA was converted to cDNA using an All-in-One First-Strand cDNA Synthesis Kit (GeneCopoeia). The cDNA was used as a template to perform qRT-PCR using the All-in-One qRT-PCR Mix (GeneCopoeia). Cycling parameters were 40 cycles of 10 min at 95°C, 20 s at 60°C, 15 s at 72°C. 16S rRNA was used as a control to estimate fold changes in the genes of interest (39). The relative gene expression levels were determined using the comparative threshold cycle method (2^−ΔΔCT^). Each qRT-PCR was performed in triplicate.

### Statistical analysis

Data points and error bars represent the mean and standard deviation of independent replicates (*N* = 3). The comparison of expression levels of major virulence genes between the Newman wild-type strain and the Δ*gltB* mutant was analyzed using the independent samples *t*-test. Differences in biofilm formation, staphyloxanthin synthesis, and expression levels of staphyloxanthin synthesis-related genes among the WT::pRAB11, Δ*gltB*::pRAB11, and Δ*gltB*::pRAB*gltB* were compared by one-way analysis of variance (ANOVA), followed by Fisher’s least significant difference (LSD) for multiple comparisons. Probit analysis was used to calculate the LD50 and 95% CIs. The statistical analysis was conducted by SPSS 13.0 software. *P* values <0.05 were considered statistically significant.

## RESULTS

### Susceptibility of the *S. aureus* Newman wild-type and the Δ*gltB* mutant to various antibiotics

Based on previous studies (11, 27, 30, 31) and our KEGG pathway analysis, to further explore the role of the glutamate metabolism pathway in the persister formation of *S. aureus*, *gltB*, which encodes a large subunit of glutamate synthase, was chosen as the target gene. The ∆*gltB* mutant strain was successfully constructed from the *S. aureus* Newman strain by homologous recombination. The susceptibility of the wild-type and Δ*gltB* mutant to ampicillin, norfloxacin, vancomycin, and rifampin was determined by measuring the MIC and MBC. Except for vancomycin, the Δ*gltB* mutant was more susceptible to ampicillin, norfloxacin, and rifampin and showed a twofold lower MIC/MBC than the parent strain (Table 1).

**TABLE 1:** MIC and MBC determination of the *Staphylococcus aureus* Newman strain and Δ*gltB* mutant for different antibiotics

Strains	MIC/MBC (μg mL^−1^)			
	Ampicillin	Norfloxacin	Vancomycin	Rifampin
*S. aureus* Newman	0.156/0.156	0.5/0.5	4/4	0.01/0.01
Δ*gltB* mutant	0.078/0.078	0.25/0.25	4/8	0.005/0.005

### The Δ*gltB* mutant had significantly reduced antibiotic tolerance at the stationary phase

**Fig 1:**
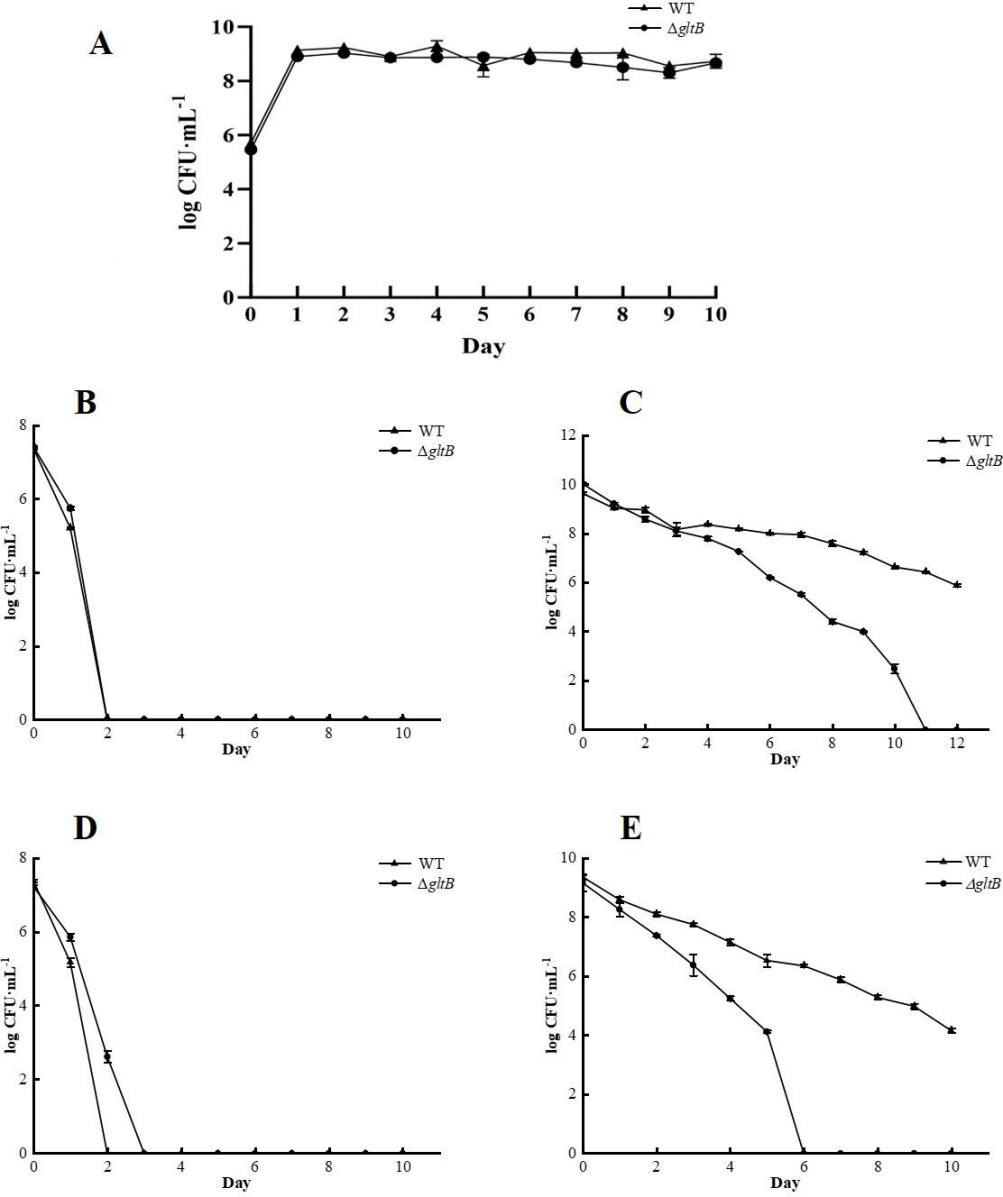
Results of growth curve in 10 days (**A**) and antibiotic exposure assay of the wild-type and the Δ*gltB* mutant in logarithmic phase (**B, D**) and stationary phase (**C, E**). (B and C) ampicillin; (D and E) norfloxacin. Data points and error bars represent the mean and SD of independent replicates (*N* = 3).

We first performed a growth curve study of 10-day to exclude the possibility that the Δ*gltB* mutant has growth defects compared to the parental strain. The results showed that the mutant had no growth defect in TSB under non-stressed conditions in 10 days (Fig. 1A). Ampicillin and norfloxacin exposure experiments were performed to investigate the differences in persister formation in cultures in the logarithmic phase (3 h) and stationary phase (24 h) between the parent strain and the Δ*gltB* mutant. The results showed that in the logarithmic phase, both the wild-type and the Δ*gltB* mutant formed fewer persister bacteria, whereas in the stationary phase, the formation of persister bacteria increased significantly (Fig. 1). The comparison of persister formation between the parent strain and the Δ*gltB* mutant showed that there was no significant difference in persister formation in cultures at the logarithmic phase between the parent strain and the Δ*gltB* mutant, and all the bacteria were killed after exposure to lethal doses of ampicillin and norfloxacin for 2 or 3 days (Fig. 1B and D). However, in stationary phase cultures, the Δ*gltB* mutant had significantly reduced antibiotic tolerance and was more easily killed, such that no viable bacteria could be detected after 11 and 6 days of ampicillin and norfloxacin exposure, respectively, while the parent strain still had more than 10^6^ CFU/mL viable bacteria remaining (Fig. 1C and E).

### Exogenous glutamate supplementation restored the persister formation ability of the Δ*gltB* mutant

**Fig 2:**
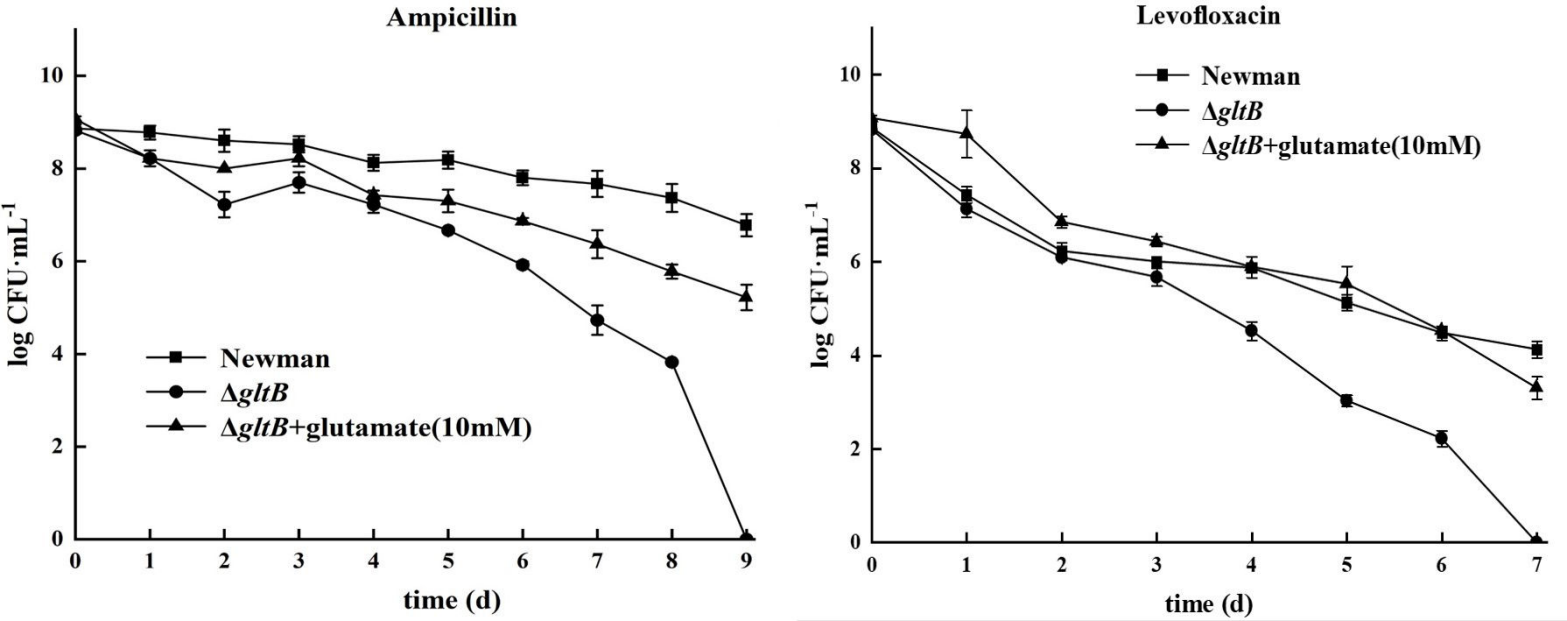
Comparison of antibiotic exposure assay of the wild-type and the Δ*gltB* mutant in TSB and TSB supplemented with glutamate. Data points and error bars represent the mean and SD of independent replicates (*N* = 3).

To further confirm the role of glutamate in persister formation in *S. aureus*, Δ*gltB* and its parent strain were inoculated into TSB containing glutamate (10 mM) for 48 h, and the cultures were used to perform ampicillin and levofloxacin exposure tests. The results showed that exposure to lethal concentrations of ampicillin resulted in no viable Δ*gltB* in TSB after 9 days of exposure, whereas there were still 10^5^ CFU/mL viable Δ*gltB* in TSB supplemented with glutamate (Fig. 2). Similar results were observed in levofloxacin exposure tests. There were no viable cells in the Δ*gltB* mutant after 7 days of exposure, whereas there were still 10^3^ CFU/mL of viable cells in the Δ*gltB* mutant supplemented with glutamate (Fig. 2). The exogenous glutamate supplementation restored the ability of persister formation of the Δ*gltB* mutant.

### The defective persistence of the Δ*gltB* mutant was restored after complementation

Using the plasmid pRAB11, the complemented strain Δ*gltB*::pRAB*gltB* and the vector control strains in the parent strain WT::pRAB11 and the mutant strain Δ*gltB*::pRAB11 were successfully constructed. The expression status of *gltB* in WT::pRAB11, Δ*gltB*::pRAB11, and Δ*gltB*::pRAB*gltB* was measured by qPCR after induction by Atc. The results showed that the expression level of *gltB* in Δ*gltB*::pRAB*gltB* (2^–∆∆CT^: 30.97 ± 3.263) was significantly higher than that in WT::pRAB11 (*P* < 0.05). No amplification was observed for the mutant vector strain Δ*gltB*::pRAB11.

**Fig 3:**
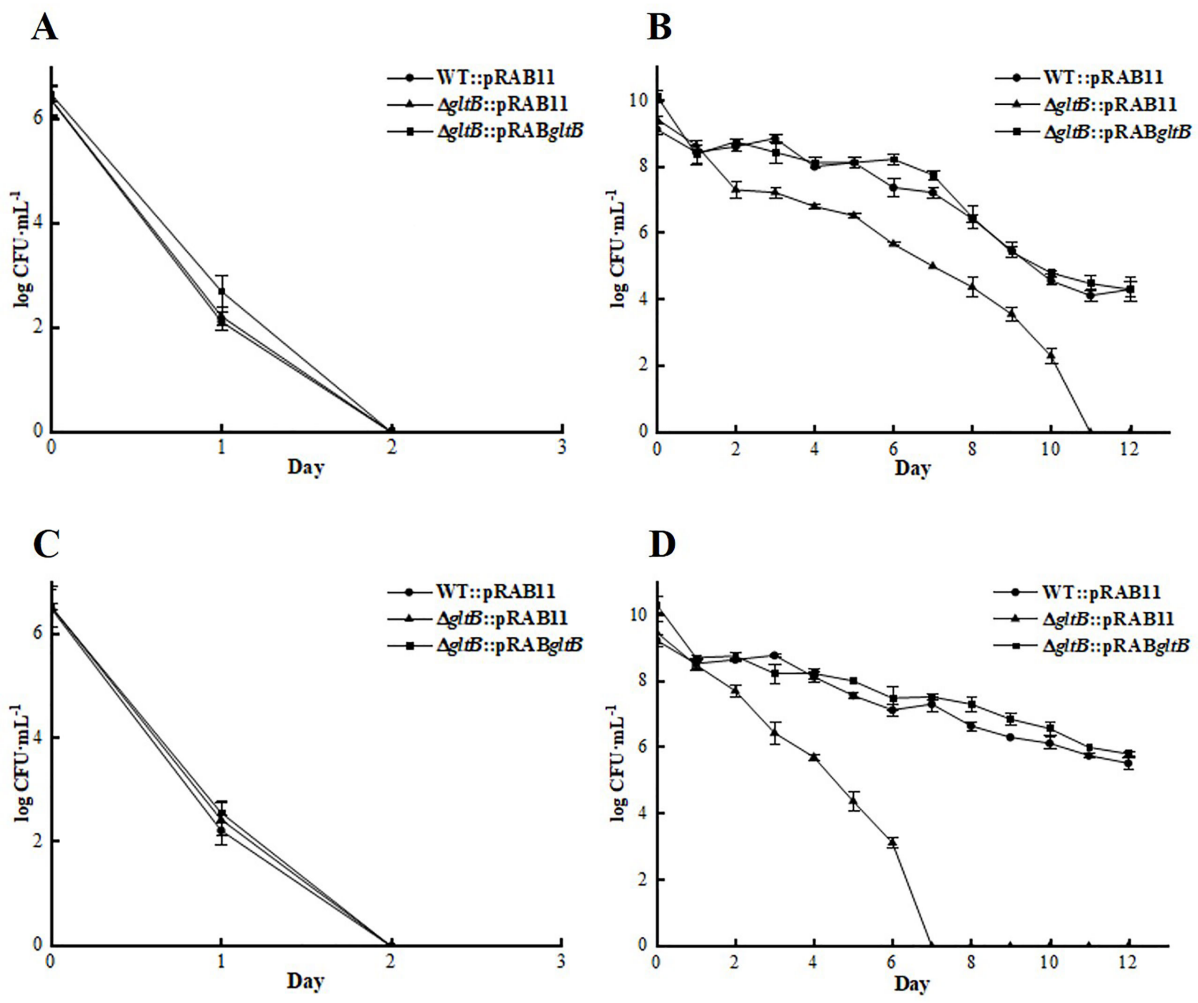
Results of antibiotic exposure assay of WT::pRAB11, Δ*gltB*::pRAB11, and Δ*gltB*::pRAB*gltB* in the logarithmic phase (**A, C**) and stationary phase (**B, D**). (A and B) ampicillin; (C and D) norfloxacin. Data points and error bars represent the mean and SD of independent replicates (*N* = 3).

Antibiotic exposure experiments were performed to confirm the role of *gltB* in persister cell formation in *S. aureus*. Similar to the uncomplemented strain, there were fewer persisters in WT::pRAB11, Δ*gltB*::pRAB11, and Δ*gltB*::pRAB*gltB* cultured in TSB containing Atc at logarithmic phase (cultured for 3 h), such that all bacteria were killed after exposure to lethal doses of ampicillin and norfloxacin for 2 days (Fig. 3A and C). However, the tolerance of the △*gltB*::pRAB11 in stationary phase (cultured 24 h) to antibiotics was significantly weaker than that of the WT::pRAB11 control, and the △*gltB*::pRAB11 was all killed by ampicillin after 11 days of exposure and norfloxacin after 7 days of exposure, while the WT::pRAB11 still had 4.12 ± 0.17 log CFU mL^−1^ bacteria surviving after 11 days of ampicillin exposure, and 7.30 ± 0.24 log CFU mL^−1^ bacteria surviving after 7 days of norfloxacin exposure. The stationary phase culture of the complemented strain △*gltB*::pRAB*gltB* restored the tolerance to ampicillin and norfloxacin (Fig. 3B and D).

### *gltB* mutant was more susceptible to heat, carbon starvation, and oxidative stress

**Fig 4:**
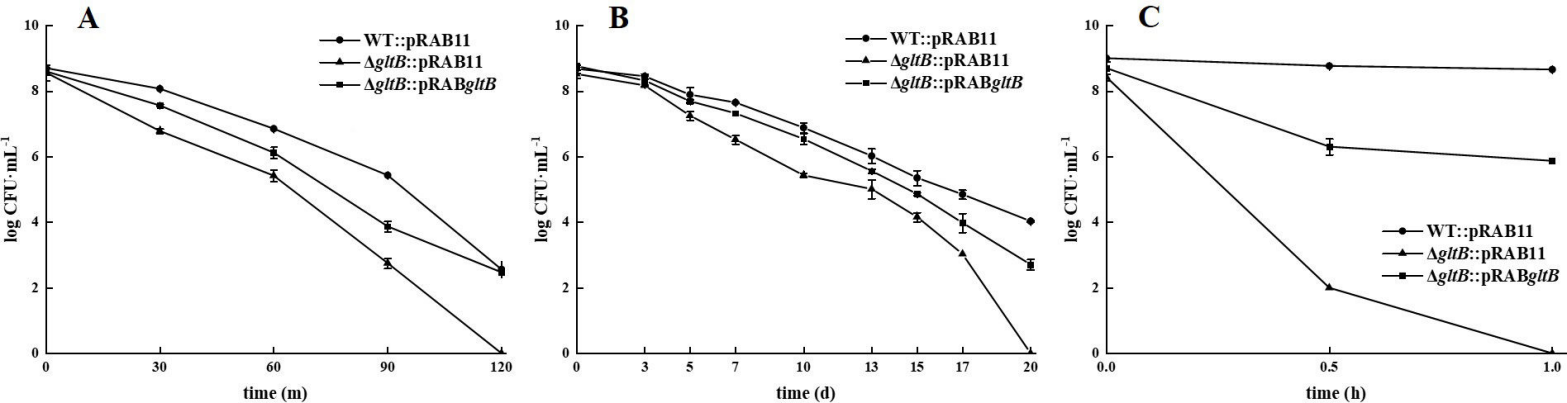
Results of WT::pRAB11, Δ*gltB*::pRAB11, and Δ*gltB*::pRAB*gltB* exposed to heat (**A**), carbon starvation (**B**), and oxidative stress (**C**). Data points and error bars represent the mean and SD of independent replicates (*N* = 3).

WT::pRAB11, Δ*gltB*::pRAB11, and Δ*gltB*::pRAB*gltB* were cultured in TSB containing Atc for 24 h. One milliliter of each culture was incubated at 56°C ± 0.1°C for 30, 60, 90, and 120 min, and the number of viable bacteria was counted at the corresponding time points. The results showed that the number of viable bacteria in Δ*gltB*::pRAB11 was lower than that in WT::pRAB11 at the corresponding time points; after 2 h of exposure, there were no viable bacteria in Δ*gltB*::pRAB11, while WT::pRAB11 still had more than 10^2^ CFU/mL. Δ*gltB*::pRAB*gltB* restored heat resistance (Fig. 4A).

To further test the resistance of the *gltB* mutant to carbon starvation, overnight WT::pRAB11, Δ*gltB*::pRAB11, and Δ*gltB*::pRAB*gltB* cultures were washed with PBS, resuspended in PBS containing Atc, and the viability of the bacteria was assessed. The results showed that Δ*gltB*::pRAB11 died in the absence of carbon sources after 20 days, while WT::pRAB11 still had 10^4^ CFU/mL viable bacteria. However, the *gltB* complemented strain Δ*gltB*::pRAB*gltB* restored resistance to carbon starvation (Fig. 4B).

Hydrogen peroxide exposure at different time points showed that the Δ*gltB*::pRAB11 mutant was much more susceptible to peroxide than WT::pRAB11. The Δ*gltB*::pRAB11 strain was completely killed after exposure for 1 h, whereas the WT::pRAB11 strain still had 10^8^ CFU/mL of surviving bacteria. Complementation of the Δ*gltB* mutant partially restored the level of peroxide susceptibility to that of WT::pRAB11 (Fig. 4C).

### The Δ*gltB* mutant had reduced ability to form staphyloxanthin

**Fig 5:**
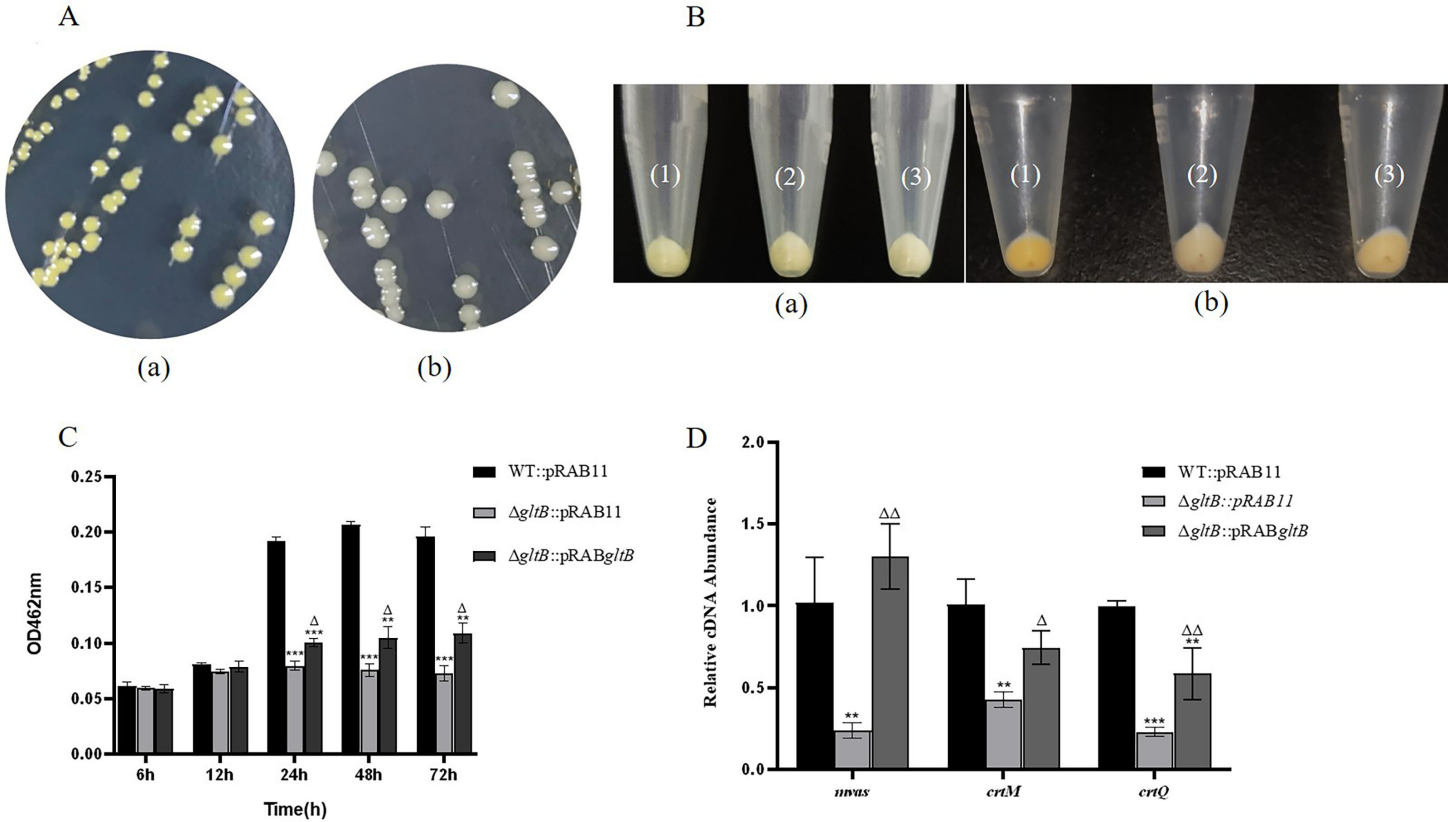
Comparison of staphyloxanthin-forming ability between the wild-type and Δ*gltB* mutant. (A) The colony of *S. aureus* wild-type (a) and Δ*gltB* mutant (b) cultured on a TSA plate. (B) The precipitate color of WT::pRAB11 (1), Δ*gltB*::pRAB11 (2), and Δ*gltB*::pRAB*gltB* (3) cultured for 6 h (a), and 48 h (b). (C) Comparison of OD462 of the methanol extracts of pigment from WT::pRAB11, Δ*gltB*::pRAB11, and Δ*gltB*::pRAB*gltB* at different time points. **P < 0.01, ***P < 0.001, compared with WT::pRAB11. Δ: *P* < 0.05, compared with Δ*gltB*::pRAB11. The error bars in the figures represent the SD (three replicates). The means of all groups were compared by one-way ANOVA. *P*-values were adjusted for multiple comparisons using the method of LSD. (D) Comparison of expression levels of major staphyloxanthin synthesis-related genes. **P < 0.01, ***P < 0.001, compared with WT::pRAB11. Δ: *P* < 0.05, ΔΔ: *P* < 0.01, compared with Δ*gltB*::pRAB11. The error bars in the figures represent the SD (three replicates). The means of all groups were compared by one-way ANOVA. *P*-values were adjusted for multiple comparisons using the method of LSD.

To analyze the effect of *gltB* allelic replacement on the growth characteristics of *S. aureus*, the wild-type and Δ*gltB* mutant were cultured on TSA for 24 h. The morphology of the colonies growing on the plate was similar, showing a round shape and a moist, smooth surface. However, the wild-type strain had a stronger ability to produce the yellow carotenoid pigment (staphyloxanthin) than the Δ*gltB* mutant (Fig. 5A).

To further analyze the effect of *gltB* allelic replacement on staphyloxanthin formation in *S. aureus*, WT::pRAB11, Δ*gltB*::pRAB11, and Δ*gltB*::pRAB*gltB* were cultured in TSB containing Atc for 6 and 48 h. While the 6 h cultures of the three bacterial strains did not show significant differences in color, after 48 h of cultivation, the WT::pRAB11 strain showed significantly more yellow color than Δ*gltB*::pRAB11. Δ*gltB*::pRAB*gltB* partially restored staphyloxanthin production (Fig. 5B).

Comparing the results at OD_462_ of methanol extracts from the three strains at different cultivation time points, there was no significant difference in staphyloxanthin content between WT::pRAB11 and Δ*gltB*::pRAB11 at 6 and 12 h. However, at 24, 48, and 72 h of culture, the staphyloxanthin production of WT::pRAB11 was significantly higher than that of Δ*gltB*::pRAB11 (*P* < 0.01). After *gltB* complementation, staphyloxanthin formation in Δ*gltB*::pRAB*gltB* was partially restored, and its staphyloxanthin production was significantly higher than that of Δ*gltB*::pRAB11 at 24, 48, and 72 h (*P* < 0.05) (Fig. 5C).

qRT-PCR revealed that the expression levels of *mvaS*, *crtM,* and *crtQ*, which are involved in the synthesis of the golden pigment, were significantly downregulated in Δ*gltB*::pRAB11 than in WT::pRAB11 (*P* < 0.01). After *gltB* complementation, the expression levels of *mvaS*, *crtM,* and *crtQ* were partially restored (Fig. 5D).

### The ability of the Δ*gltB* mutant to coagulate plasma was significantly weakened

**Fig 6:**
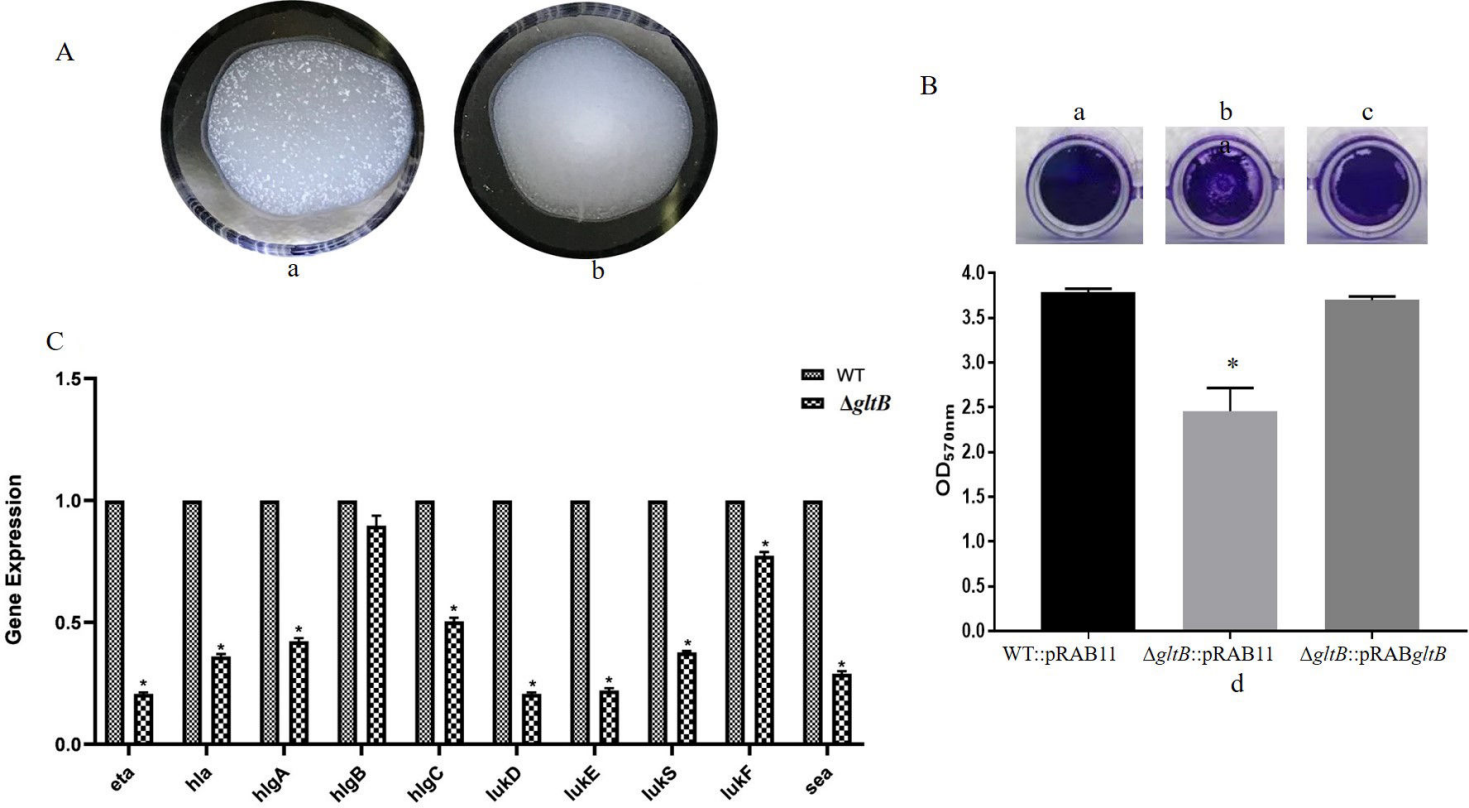
Comparison of plasma coagulation, biofilm-forming ability, and expression levels (2^−∆∆CT^) of major virulence genes between the wild-type and the *∆gltB* mutant. (A) Plasma coagulation ability. (a) Normal coagulation with wild type. (b) Defective coagulation with the *∆gltB* mutant; (B) biofilms formed by WT::pRAB11 (Ba), Δ*gltB*::pRAB11 (Bb), and Δ*gltB*::pRAB*gltB* (Bc). Comparison of OD_570_ nm of the measured wells after 33% acetic acid was added. *P < 0.05 (Bd); (C) expression levels of major virulence genes. *P < 0.05. The error bars in the figures represent the SD (three replicates). The means of all groups were compared by an independent samples *t*-test.

*S. aureus* can produce staphylocoagulase, which can activate prothrombin to generate fibrin for the formation of *S. aureus*-fibrin-platelet microaggregates and for homing of the bacteria to the vascular wall under flow (40). Coagulase is an important virulence factor of *S. aureus*. To determine the effect of the *gltB* allelic replacement on the ability of *S. aureus* to coagulate plasma, overnight cultures of the parent strain and the ∆*gltB* mutant were mixed with rabbit plasma containing sodium citrate and incubated for 10 min at room temperature. The parent strain had normal coagulation, while the Δ*gltB* mutant had a defect in coagulation (Fig. 6A).

### *gltB* deletion inhibited biofilm formation in *S. aureus*

To further explore the relationship between *gltB* and biofilm formation in *S. aureus*, WT::pRAB11, Δ*gltB*::pRAB11, and Δ*gltB*::pRAB*gltB* were cultured in 96-well plates for 24 h and then stained with crystal violet, and the OD_570_ nm was determined. The results showed that the biofilm-forming ability of Δ*gltB*::pRAB11 was significantly lower than that of WT::pRAB11, and the biofilm formation ability of the complemented strain Δ*gltB*::pRAB*gltB* was restored (Fig. 6B) (*P* < 0.05).

### The Δ*gltB* mutant had reduced virulence in mice

The LD50 of *S. aureus* Newman wild-type and the Δ*gltB* mutant in BALB/c mice were assessed by intraperitoneal injection. The number of deaths and survivors in each group is shown in Table 2.

**TABLE 2:** LD50 determination (i.p.) of *Staphylococcus aureus* Newman strain and Δ*gltB* mutant in BALB/c Mice^*a*^

Group	Dose (CFU/mL)	Number of animals	Number of deaths (k)	Number of survivors	Mortality rate (%)
*S. aureus* Newman strain					
1	5.33 × 10^10^	5	5	0	100
2	3.33 × 10^10^	5	5	0	100
3	1.67 × 10^10^	5	5	0	100
4	7.67 × 10^9^	5	5	0	100
5	3.67 × 10^9^	5	3	2	60
6	1.67 × 10^9^	5	2	3	40
7	5.33 × 10^8^	5	0	5	0
Δ*gltB* mutant					
1	6.33 × 10^10^	5	5	0	100
2	3.33 × 10^10^	5	5	0	100
3	1.33 × 10^10^	5	3	2	60
4	7.33 × 10^9^	5	1	4	20
5	4.33 × 10^9^	5	0	5	0
6	1.67 × 10^9^	5	0	5	0
7	6.67 × 10^8^	5	0	5	0

^*a*^ i.p., intraperitoneal injection.

Calculated LD50 (Probit analysis): LD50 of *S. aureus* Newman strain = 2.39 × 10^9^ CFU/mL (95% CI: 9.99 × 10^8^–4.42 × 10^9^); LD50 of Δ*gltB* mutant = 1.14 × 10^10^ CFU/mL (95% CI: 7.29 × 10^9^–2.75 × 10^10^). The results demonstrate that the LD50 of the Newman wild type is significantly lower than that of the Δ*gltB* mutant.

### The expression levels of major virulence genes in the Δ*gltB* were decreased

To further analyze the mechanism of the effect of the *gltB* allelic replacement on the virulence of *S. aureus*, the expression levels of major virulence genes, including *eta*, *hla*, *hlgA*, *hlgB*, *hlgC*, *lukD*, *lukE*, *lukS*, *lukF*, and *sea* in the parent strain and the Δ*gltB* mutant were determined by qRT-PCR. Except for *hlgB*, the expression levels of the major virulence genes, including *eta*, *hla*, *hlgA*, *hlgC*, *lukD*, *lukE*, *lukS*, *lukF*, and *sea*, were significantly higher in the parent strain than in the ∆*gltB* mutant (*P* < 0.05) (Fig. 6C).

## DISCUSSION

The mechanism of persister cell formation by *S. aureus* is not well understood. This study investigated the effect of glutamate metabolism on persister cell formation in *S. aureus*. We found that after knocking out *gltB*, a key gene for glutamate synthesis, the *gltB* mutant had a decreased ability to form persisters in the stationary phase, as the mutant could be easily killed by antibiotics and showed increased susceptibility to various stresses, including heat, carbon starvation, and oxidants. These defects can be restored by adding exogenous glutamate to the medium or by complementation with *gltB*. Meanwhile, this study also found that decreased synthesis of glutamate in the *gltB* mutant significantly affected the virulence of *S. aureus*, as shown by its increased LD50 in mice and downregulated expression of related virulence genes. Furthermore, the ability of the *gltB* mutant to coagulate plasma, produce staphyloxanthin, and form biofilms was significantly reduced compared to that of the wild strain. Thus, our findings provide strong evidence that glutamate synthesis is involved in persister cell formation and virulence in *S. aureus*.

Glutamate is an important molecule in bacterial cells and is involved in several metabolic processes, including protein synthesis, glycolysis, gluconeogenesis, and the TCA cycle, linking nitrogen and carbon metabolisms (41). The results of this study demonstrate that a deficiency in glutamate synthesis increases the susceptibility of the mutant strain to ampicillin, norfloxacin, and rifampicin. Although both ampicillin and vancomycin act on the bacterial cell wall, their target sites and mechanisms exhibit subtle differences. Ampicillin primarily binds to penicillin-binding proteins, thereby inhibiting peptidoglycan synthesis (42). In contrast, vancomycin binds directly to peptidoglycan precursors, specifically targeting the d-Ala-d-Ala terminus, which blocks peptidoglycan assembly (43). In this study, the impact of glutamate synthesis deficiency in the mutant strain on the target specificity and mechanisms of ampicillin and vancomycin at the cell wall requires further investigation in future research.

**Fig 7:**
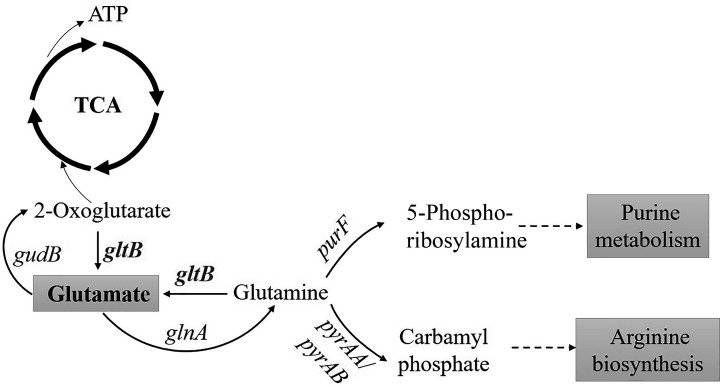
Pathways that indicate how *gltB* is connected to TCA cycle, purine, and arginine metabolism to contribute to antibiotic tolerance in *S. aureus.*

Glutamate metabolism is linked to the TCA cycle, purine metabolism, arginine biosynthesis, and other important metabolic processes in *S. aureus. gltB* is a key gene that encodes the large subunit of glutamate synthesis, which catalyzes the conversion of 2-oxoglutarate and glutamine into glutamate, which is a general amino group donor in the metabolism of *S. aureus*. Glutamate can be converted into 2-oxoglutarate by glutamate dehydrogenase (encoded by *gudB*) and enters the TCA cycle to generate energy. Glutamine synthetase (encoded by *glnA*) catalyzes glutamate to glutamine (44), which is involved in purine metabolism and arginine biosynthesis through the action of amidophosphoribosyltransferase (encoded by *purF*) (45–47) and carbamyl phosphate synthetase (encoded by *pyrAA*/*pyrAB*). These analyses highlight the pivotal role of glutamate metabolism in the regulation of the above metabolic pathways, which were confirmed to participate in persister formation in *S. aureus* in this study. Therefore, the pathway that converts 2-oxoglutarate to glutamate would be blocked, which would facilitate 2-oxoglutarate to enter the TCA cycle and generate ATP (48), resulting in a decrease in persister formation in the *gltB* mutant when the *gltB* gene of *S. aureu*s was knocked out. At the same time, due to the reduced synthesis of glutamate, the produced cascade reaction affects purine metabolism and arginine biosynthesis, leading to decreased antibiotic tolerance in bacteria (27, 31). A diagram linking the above pathways to explain how *gltB* is involved in persister cell formation in *S. aureus* is shown in Fig. 7. Our previous study found that *purN* affects persister cell formation in *S. aureus* via *gltB* (32). Furthermore, the deletion of *gltB* increased the susceptibility of *S. aureus* to ampicillin, norfloxacin, and rifampicin, as shown by a twofold decrease in MIC/MBC compared with the parent strain, which is consistent with previous results in the case of persister genes *phoU* and *sucB* involved in persister formation in *E. coli* (10, 49). The mechanism by which glutamate metabolism participates in the above metabolism to affect the persister formation in *S. aureus* needs further study in the future.

A link between glutamate metabolism and persister formation has been previously reported. By screening a transposon mutant library, Yee et al. found that the *gltT* (encoding protein/sodium glutamate synchronous protein) mutation in *S. aureus* USA300 resulted in reduced tolerance to heat ramps, acetic acid, and antibiotics (34). Li et al. reported that the *gltI* (encoding glutamate/aspartate ABC transporter ATP-binding subunit) mutant of *E. coli* failed to form tosufloxacin-tolerant persisters (33). Decarboxylation of glutamate to γ-aminobutyrate is important for acid resistance in *E. coli* (41). The *hipA2*-encoded kinase in *Caulobacter* induces bacterial persistence by disturbing the intracellular tryptophan-glutamine balance (50). In addition, it has been shown that deletion of *fis,* which regulates glutamate metabolism, can significantly reduce persister formation in *Salmonella* typhi (51). These observations are consistent with our finding that glutamate synthase mutations could affect persister formation in *S. aureus* in this study.

The production of virulence factors and biofilm formation is closely related to the virulence of *S. aureus*. In the present study, the virulence of the *gltB* mutant was significantly reduced. The reason for the decrease in virulence may be related to the significantly reduced expression of major virulence factor genes (*eta, hla, hlgA, hlgC, lukD, lukE, lukS, lukF,* and *sea*) and the biofilm formation ability of the *gltB* mutant. At present, there are few reports on the direct involvement of glutamate metabolism in the expression of virulence factors in *S. aureus*, which requires further research. Staphylocoagulase is an important virulence factor in *S. aureus* (40). The results of this study showed that allelic replacement of *gltB* resulted in a significant reduction in the ability of *S. aureus* to coagulate plasma, which is consistent with our result that the virulence of the Δ*gltB* mutant in BALB/c mice was lower than that of the parent strain. Several studies have reported the involvement of glutamate metabolism in bacterial biofilm formation. Vudhya et al. discovered that increased glutamine synthase activity in *S. aureus* correlates with elevated biofilm formation (44). Kimura et al. found that glutamate synthase is related to *Bacillus subtilis* biofilm formation. The Δ*gltA* mutant of *B. subtilis* can accumulate citrate, causing an iron shortage due to chelation, leading to defective biofilm formation in a medium containing glycerol (52). Hassanov et al. demonstrated that utilization of glutamine or glutamate as a nitrogen source is important for the development of *B. subtilis* biofilms (53). Our work found that the allelic replacement of *gltB* in *S. aureus* significantly reduced biofilm formation, which is consistent with previously reported results.

The major pigment in *S. aureus* is staphyloxanthin, which has numerous conjugated double bonds and acts as a virulence factor in *S. aureus* because of its antioxidant activity and neutrophil resistance (54–57). In this study, we found that both yellow staphyloxanthin formation and the antioxidant ability of the *gltB* mutant were significantly reduced, which could also be the reason for the reduced virulence of the ∆*gltB* mutant. An operon, *crtOPQMN,* and a *sigma(B*)-dependent promoter were upstream of *crtO* involved in staphyloxanthin biosynthesis (54, 58). We found that the expression levels of *mvaS*, *crtM,* and *crtQ*, which are involved in staphyloxanthin synthesis, were significantly downregulated. SigB, a global stress response regulator in *S. aureus*, regulates staphyloxanthin biosynthesis (59). By screening a collection of *S. aureus* transposon mutants, Lan et al. found that mutations in purine metabolism genes (*purN, purH, purD,* and *purA*) enhanced pigment formation in *S. aureus*, and this process is likely caused by increased expression of *sigB* (60). Glutamate is the main component that provides a nitrogen source in bacteria and is closely related to purine biosynthesis. Our previous study showed that overexpression of *purN* could upregulate the expression of *gltB*, indicating a close relationship between *gltB* and purine metabolism (32). Further studies are needed to determine whether *gltB* regulates the formation of staphyloxanthin by affecting glutamate production or purine metabolism and then affecting the expression of *sigB*.

In summary, glutamate metabolism plays a pivotal role in linking several important metabolic processes associated with persister formation in *S. aureus*. This study revealed that *gltB* regulates persister cells and biofilm formation and virulence in *S. aureus*. Our work provides new insights into the mechanisms of persister formation and virulence and has implications for developing new anti-persister and anti-virulence drugs for more effective treatment of *S. aureus* persistent infections.

## SUPPLEMENTAL MATERIAL

Tables S1 spectrum.00511-25-s0001.docx Sequences of the primers used in this study.

ASM does not own the copyrights to Supplemental Material that may be linked to, or accessed through, an article. The authors have granted ASM a non-exclusive, world-wide license to publish the Supplemental Material files. Please contact the corresponding author directly for reuse.

## References

[1] Bigger JW. 1944. Treatment of staphylococcal infections with penicillin by intermittent sterilisation. Lancet 244:497–500. doi:10.1016/S0140-6736(00)74210-3

[2] Lewis K. 2010. Persister cells. Annu Rev Microbiol 64:357–372. doi:10.1146/annurev.micro.112408.134306 20528688

[3] Zhang Y. 2014. Persisters, persistent infections and the Yin-Yang model. Emerg Microbes Infect 3:1–10. doi:10.1038/emi.2014.3 PMC3913823 26038493

[4] Balaban NQ, Merrin J, Chait R, Kowalik L, Leibler S. 2004. Bacterial persistence as a phenotypic switch. Science 305:1622–1625. doi:10.1126/science.1099390 15308767

[5] Kwan BW, Chowdhury N, Wood TK. 2015. Combatting bacterial infections by killing persister cells with mitomycin C. Environ Microbiol 17:4406–4414. doi:10.1111/1462-2920.12873 25858802

[6] Costerton JW, Stewart PS, Greenberg EP. 1999. Bacterial biofilms: a common cause of persistent infections. Science 284:1318–1322. doi:10.1126/science.284.5418.1318 10334980

[7] Hurdle JG, Deshpande A. 2018. Bacterial persister cells tackled. Nature 556:40–41. doi:10.1038/d41586-018-03440-w 29620744

[8] Defraine V, Fauvart M, Michiels J. 2018. Fighting bacterial persistence: current and emerging anti-persister strategies and therapeutics. Drug Resist Updat 38:12–26. doi:10.1016/j.drup.2018.03.002 29857815

[9] Barrett TC, Mok WWK, Murawski AM, Brynildsen MP. 2019. Enhanced antibiotic resistance development from fluoroquinolone persisters after a single exposure to antibiotic. Nat Commun 10:1177. doi:10.1038/s41467-019-09058-4 30862812 PMC6414640

[10] Ma C, Sim S, Shi W, Du L, Xing D, Zhang Y. 2010. Energy production genes sucB and ubiF are involved in persister survival and tolerance to multiple antibiotics and stresses in Escherichia coli. FEMS Microbiol Lett 303:33–40. doi:10.1111/j.1574-6968.2009.01857.x 20041955

[11] Wang W, Chen J, Chen G, Du X, Cui P, Wu J, Zhao J, Wu N, Zhang W, Li M, Zhang Y. 2015. Transposon mutagenesis identifies novel genes associated with Staphylococcus aureus persister formation. Front Microbiol 6:1437. doi:10.3389/fmicb.2015.01437 26779120 PMC4689057

[12] Wang Y, Bojer MS, George SE, Wang Z, Jensen PR, Wolz C, Ingmer H. 2018. Inactivation of TCA cycle enhances Staphylococcus aureus persister cell formation in stationary phase. Sci Rep 8:10849. doi:10.1038/s41598-018-29123-0 30022089 PMC6052003

[13] Conlon BP, Rowe SE, Gandt AB, Nuxoll AS, Donegan NP, Zalis EA, Clair G, Adkins JN, Cheung AL, Lewis K. 2016. Persister formation in Staphylococcus aureus is associated with ATP depletion. Nat Microbiol 1:16051. doi:10.1038/nmicrobiol.2016.51 PMC4932909 27572649

[14] Han J, He L, Shi W, Xu X, Wang S, Zhang S, Zhang Y. 2014. Glycerol uptake is important for L-form formation and persistence in Staphylococcus aureus. PLoS One 9:e108325. doi:10.1371/journal.pone.0108325 25251561 PMC4177120

[15] Page R, Peti W. 2016. Toxin-antitoxin systems in bacterial growth arrest and persistence. Nat Chem Biol 12:208–214. doi:10.1038/nchembio.2044 26991085

[16] Svenningsen MS, Veress A, Harms A, Mitarai N, Semsey S. 2019. Birth and resuscitation of (p)ppGpp induced antibiotic tolerant persister cells. Sci Rep 9:6056. doi:10.1038/s41598-019-42403-7 30988388 PMC6465370

[17] Yamanaka K, Zheng W, Crooke E, Wang YH, Inouye M. 2001. CspD, a novel DNA replication inhibitor induced during the stationary phase in Escherichia coli. Mol Microbiol 39:1572–1584. doi:10.1046/j.1365-2958.2001.02345.x 11260474

[18] Kwan BW, Valenta JA, Benedik MJ, Wood TK. 2013. Arrested protein synthesis increases persister-like cell formation. Antimicrob Agents Chemother 57:1468–1473. doi:10.1128/AAC.02135-12 23295927 PMC3591907

[19] Leszczynska D, Matuszewska E, Kuczynska-Wisnik D, Furmanek-Blaszk B, Laskowska E. 2013. The formation of persister cells in stationary-phase cultures of Escherichia coli is associated with the aggregation of endogenous proteins. PLoS ONE 8:e54737. doi:10.1371/journal.pone.0054737 23358116 PMC3554633

[20] Shan Y, Lazinski D, Rowe S, Camilli A, Lewis K. 2015. Genetic basis of persister tolerance to aminoglycosides in Escherichia coli. mBio 6:e00078-15. doi:10.1128/mBio.00078-15 25852159 PMC4453570

[21] Huemer M, Mairpady Shambat S, Bergada-Pijuan J, Söderholm S, Boumasmoud M, Vulin C, Gómez-Mejia A, Antelo Varela M, Tripathi V, Götschi S, Marques Maggio E, Hasse B, Brugger SD, Bumann D, Schuepbach RA, Zinkernagel AS. 2021. Molecular reprogramming and phenotype switching in Staphylococcus aureus lead to high antibiotic persistence and affect therapy success. Proc Natl Acad Sci USA 118:e2014920118. doi:10.1073/pnas.2014920118 33574060 PMC7896289

[22] Ikuta KS, Swetschinski LR, Robles Aguilar G, Sharara F, Mestrovic T, Gray AP, Davis Weaver N, Wool EE, Han C, Gershberg Hayoon A, et al.. 2022. Global mortality associated with 33 bacterial pathogens in 2019: a systematic analysis for the Global Burden of Disease Study 2019. Lancet 400:2221–2248. doi:10.1016/S0140-6736(22)02185-7 36423648 PMC9763654

[23] Xu T, Wang X-Y, Cui P, Zhang Y-M, Zhang W-H, Zhang Y. 2017. The Agr quorum sensing system represses persister formation through regulation of phenol soluble modulins in Staphylococcus aureus. Front Microbiol 8:2189. doi:10.3389/fmicb.2017.02189 29163457 PMC5681930

[24] Bojer MS, Lindemose S, Vestergaard M, Ingmer H. 2018. Quorum sensing-regulated phenol-soluble modulins limit persister cell populations in Staphylococcus aureus. Front Microbiol 9:255. doi:10.3389/fmicb.2018.00255 29515541 PMC5826201

[25] Pandey S, Sahukhal GS, Elasri MO. 2019. The msaABCR operon regulates the response to oxidative stress in Staphylococcus aureus. J Bacteriol 201:e00417-19. doi:10.1128/JB.00417-19 31427392 PMC6779459

[26] Richards RL, Haigh RD, Pascoe B, Sheppard SK, Price F, Jenkins D, Rajakumar K, Morrissey JA. 2015. Persistent Staphylococcus aureus isolates from two independent cases of bacteremia display increased bacterial fitness and novel immune evasion phenotypes. Infect Immun 83:3311–3324. doi:10.1128/IAI.00255-15 26056388 PMC4496624

[27] Yee R, Cui P, Shi W, Feng J, Wang J, Zhang Y. 2020. Identification of a novel gene argJ involved in arginine biosynthesis critical for persister formation in Staphylococcus aureus. Discov Med 29:65–77. 32598864

[28] Springer MT, Singh VK, Cheung AL, Donegan NP, Chamberlain NR. 2016. Effect of clpP and clpC deletion on persister cell number in Staphylococcus aureus. J Med Microbiol 65:848–857. doi:10.1099/jmm.0.000304 27375177

[29] Xu T, Han J, Zhang J, Chen J, Wu N, Zhang W, Zhang Y. 2016. Absence of protoheme IX farnesyltransferase CtaB causes virulence attenuation but enhances pigment production and persister survival in MRSA. Front Microbiol 7:1625. doi:10.3389/fmicb.2016.01625 27822202 PMC5076432

[30] Zalis EA, Nuxoll AS, Manuse S, Clair G, Radlinski LC, Conlon BP, Adkins J, Lewis K. 2019. Stochastic variation in expression of the tricarboxylic acid cycle produces persister cells. mBio 10:e01930-19. doi:10.1128/mBio.01930-19 31530676 PMC6751062

[31] Yee R, Cui P, Shi W, Feng J, Zhang Y. 2015. Genetic screen reveals the role of purine metabolism in Staphylococcus aureus persistence to rifampicin. Antibiotics (Basel) 4:627–642. doi:10.3390/antibiotics4040627 27025643 PMC4790316

[32] Peng Q, Guo L, Dong Y, Bao T, Wang H, Xu T, Zhang Y, Han J. 2022. PurN is involved in antibiotic tolerance and virulence in Staphylococcus aureus. Antibiotics (Basel) 11:1702. doi:10.3390/antibiotics11121702 36551359 PMC9774800

[33] Li T, Wang J, Cao Q, Li F, Han J, Zhu B, Zhang Y, Niu H. 2020. Identification of novel genes involved in Escherichia coli persistence to tosufloxacin. Front Cell Infect Microbiol 10:581986. doi:10.3389/fcimb.2020.581986 33117736 PMC7561378

[34] Yee R, Feng J, Wang J, Chen J, Zhang Y. 2019. Identification of genes regulating cell death In Staphylococcus aureus. Front Microbiol 10:2199. doi:10.3389/fmicb.2019.02199 31632363 PMC6779855

[35] Halsey CR, Lei S, Wax JK, Lehman MK, Nuxoll AS, Steinke L, Sadykov M, Powers R, Fey PD. 2017. Amino acid catabolism in Staphylococcus aureus and the function of carbon catabolite repression. mBio 8:e01434-16. doi:10.1128/mBio.01434-16 28196956 PMC5312079

[36] Reitzer L. 2003. Nitrogen assimilation and global regulation in Escherichia coli. Annu Rev Microbiol 57:155–176. doi:10.1146/annurev.micro.57.030502.090820 12730324

[37] Bae T, Schneewind O. 2006. Allelic replacement in Staphylococcus aureus with inducible counter-selection. Plasmid 55:58–63. doi:10.1016/j.plasmid.2005.05.005 16051359

[38] Kuehl R, Al-Bataineh S, Gordon O, Luginbuehl R, Otto M, Textor M, Landmann R. 2009. Furanone at subinhibitory concentrations enhances staphylococcal biofilm formation by luxS repression. Antimicrob Agents Chemother 53:4159–4166. doi:10.1128/AAC.01704-08 19620329 PMC2764226

[39] Luong TT, Dunman PM, Murphy E, Projan SJ, Lee CY. 2006. Transcription profiling of the mgrA regulon in Staphylococcus aureus. J Bacteriol 188:1899–1910. doi:10.1128/JB.188.5.1899-1910.2006 16484201 PMC1426550

[40] Peetermans M, Verhamme P, Vanassche T. 2015. Coagulase activity by Staphylococcus aureus: a potential target for therapy? Semin Thromb Hemost 41:433–444. doi:10.1055/s-0035-1549849 25973589

[41] Feehily C, Karatzas KAG. 2013. Role of glutamate metabolism in bacterial responses towards acid and other stresses. J Appl Microbiol 114:11–24. doi:10.1111/j.1365-2672.2012.05434.x 22924898

[42] Zapun A, Contreras-Martel C, Vernet T. 2008. Penicillin-binding proteins and beta-lactam resistance. FEMS Microbiol Rev 32:361–385. doi:10.1111/j.1574-6976.2007.00095.x 18248419

[43] Kahne D, Leimkuhler C, Lu W, Walsh C. 2005. Glycopeptide and lipoglycopeptide antibiotics. Chem Rev 105:425–448. doi:10.1021/cr030103a 15700951

[44] Vudhya Gowrisankar Y, Manne Mudhu S, Pasupuleti SK, Suthi S, Chaudhury A, Sarma PVGK. 2021. Staphylococcus aureus grown in anaerobic conditions exhibits elevated glutamine biosynthesis and biofilm units. Can J Microbiol 67:323–331. doi:10.1139/cjm-2020-0434 33136443

[45] Li L, Abdelhady W, Donegan NP, Seidl K, Cheung A, Zhou Y-F, Yeaman MR, Bayer AS, Xiong YQ. 2018. Role of purine biosynthesis in persistent methicillin-resistant Staphylococcus aureus infection. J Infect Dis 218:1367–1377. doi:10.1093/infdis/jiy340 29868791 PMC6151072

[46] Lu CD. 2006. Pathways and regulation of bacterial arginine metabolism and perspectives for obtaining arginine overproducing strains. Appl Microbiol Biotechnol 70:261–272. doi:10.1007/s00253-005-0308-z 16432742

[47] Nuxoll AS, Halouska SM, Sadykov MR, Hanke ML, Bayles KW, Kielian T, Powers R, Fey PD. 2012. CcpA regulates arginine biosynthesis in Staphylococcus aureus through repression of proline catabolism. PLoS Pathog 8:e1003033. doi:10.1371/journal.ppat.1003033 23209408 PMC3510247

[48] Muro-Pastor MI, Reyes JC, Florencio FJ. 2005. Ammonium assimilation in cyanobacteria. Photosynth Res 83:135–150. doi:10.1007/s11120-004-2082-7 16143848

[49] Li Y, Zhang Y. 2007. PhoU is a persistence switch involved in persister formation and tolerance to multiple antibiotics and stresses in Escherichia coli. Antimicrob Agents Chemother 51:2092–2099. doi:10.1128/AAC.00052-07 17420206 PMC1891003

[50] Zhou X, Eckart MR, Shapiro L. 2021. A bacterial toxin perturbs intracellular amino acid balance to induce persistence. mBio 12:e03020-20. doi:10.1128/mBio.03020-20 PMC8545095 33622732

[51] Yan D, Zhang Q, Fu Q, Sun M, Huang X. 2021. Disruption of Fis reduces bacterial persister formation by regulating glutamate metabolism in Salmonella. Microb Pathog 152:104651. doi:10.1016/j.micpath.2020.104651 33249164

[52] Kimura T, Kobayashi K. 2020. Role of glutamate synthase in biofilm formation by Bacillus subtilis. J Bacteriol 202:e00120-20. doi:10.1128/JB.00120-20 32393519 PMC7317036

[53] Hassanov T, Karunker I, Steinberg N, Erez A, Kolodkin-Gal I. 2018. Novel antibiofilm chemotherapies target nitrogen from glutamate and glutamine. Sci Rep 8:7097. doi:10.1038/s41598-018-25401-z 29740028 PMC5940852

[54] Xue L, Chen YY, Yan Z, Lu W, Wan D, Zhu H. 2019. Staphyloxanthin: a potential target for antivirulence therapy. Infect Drug Resist 12:2151–2160. doi:10.2147/IDR.S193649 31410034 PMC6647007

[55] El-Agamey A, Lowe GM, McGarvey DJ, Mortensen A, Phillip DM, Truscott TG, Young AJ. 2004. Carotenoid radical chemistry and antioxidant/pro-oxidant properties. Arch Biochem Biophys 430:37–48. doi:10.1016/j.abb.2004.03.007 15325910

[56] Liu GY, Essex A, Buchanan JT, Datta V, Hoffman HM, Bastian JF, Fierer J, Nizet V. 2005. Staphylococcus aureus golden pigment impairs neutrophil killing and promotes virulence through its antioxidant activity. J Exp Med 202:209–215. doi:10.1084/jem.20050846 16009720 PMC2213009

[57] Cueno ME, Imai K. 2018. Network analytics approach towards identifying potential antivirulence drug targets within the Staphylococcus aureus staphyloxanthin biosynthetic network. Arch Biochem Biophys 645:81–86. doi:10.1016/j.abb.2018.03.010 29551420

[58] Pelz A, Wieland KP, Putzbach K, Hentschel P, Albert K, Götz F. 2005. Structure and biosynthesis of staphyloxanthin from Staphylococcus aureus. J Biol Chem 280:32493–32498. doi:10.1074/jbc.M505070200 16020541

[59] Pannu MK, Hudman DA, Sargentini NJ, Singh VK. 2019. Role of SigB and staphyloxanthin in radiation survival of Staphylococcus aureus. Curr Microbiol 76:70–77. doi:10.1007/s00284-018-1586-x 30353215

[60] Lan L, Cheng A, Dunman PM, Missiakas D, He C. 2010. Golden pigment production and virulence gene expression are affected by metabolisms in Staphylococcus aureus. J Bacteriol 192:3068–3077. doi:10.1128/JB.00928-09 20400547 PMC2901709

