# Supplementary material for: gltB encoding glutamate synthase is involved in persister and biofilm formation and virulence in Staphylococcus aureus
Source: Microbiol Spectr. 2025 Aug 12;13(9):e00511-25. doi: 10.1128/spectrum.00511-25 (PMC12403659; doi:10.1128/spectrum.00511-25)
Supplement: Tables S1 — Sequences of the primers used in this study. [file spectrum.00511-25-s0001.docx]

Supplementary Materials

Table S1 Sequences of the primers used in this study

| **Primer name** | **Sequences (5’→3’)** | **Source or reference** |
| --- | --- | --- |
| gltB-uf-KpnI | GGGGTACCTCATCATTGATAGCATCGAATTACT | This study |
| gltB-ur | ATCGTATCTCCCCCTTTCCGATGCACACCCCTTTTTTA | This study |
| gltB-df | TAAAAAAGGGGTGTGCATCGGAAAGGGGGAGATACGAT | This study |
| gltB-dr-EcoRI | CGGAATTCGGTCCTTCTTTCATGACCATA | This study |
| gltB-f | gaagatctatgcacaatgagaaattaattaaag | This study |
| gltB-r | cggaattcttaatacacaacggctggtttatgtttagc | This study |
| q-*gltB* f | TTGCGAAGACAAGAGGGT | This study |
| q-*gltB* r | CTAAACCAATCTCCCAAGGA | This study |
| eta-F | GCACCCGTTAGCGGATATT | This study |
| eta-R | GTCGATGTGTTCGGTTTGATTG | This study |
| hla-F | AGATTCTTGGAACCCGGTATATG | This study |
| hla-R | GCTTTGTTAGGATCAAGGAAGTTATC | This study |
| hlgA-F | TCCAATCAGCGCCATCAA | This study |
| hlgA-R | ACACCTTTAGAGTTCTGACTTTCT | This study |
| hlgB-F | AATGTTGGCTGGGGAGT | This study |
| hlgB-R | CGCTATGAAGTTTTGGC | This study |
| hlgC-F | CGCCACTGAATCAGGTCAAA | This study |
| hlgC-R | ACTCACTGTCTGGAACGAAATAA | This study |
| lukD-F | GGCAGCCGGAAACATTAATTC | This study |
| lukD-R | GGTGCATAGTCAACAACATTTACA | This study |
| lukE-F | GGACTGACGACTAAAGATCCAAA | This study |
| lukE-R | AGATGGTGCTGACTGGAAATTA | This study |
| lukS-F | AGCTCAGGTGGTAAATTCGATTC | This study |
| lukS-R | GACCAGTGTACATGCCAGTTATT | This study |
| sea-F | CCCTAACGTGGACAACAAGTC | This study |
| sea-R | CTAAAGCTGCTCCCTGCAAT | This study |
| lukF-F | TCAGACACAGTTACAGGCA | This study |
| lukF-R | GTTGGAAAGTAGAAGCACA | This study |
| 16S-F | CGTGCTACAATGGACAATACAAA | * |
| 16S-R | ATCTACGATTACTAGCGATTCCA | * |

*. Thanh T Luong, Paul M Dunman, Ellen Murphy, Steven J Projan, Chia Y Lee. Transcription Profiling of the mgrA Regulon in *Staphylococcus aureus*. J Bacteriol. 2006;188(5):1899-910.
